# Designing and Testing an Inventory for Measuring Social Media Competency of Certified Health Education Specialists

**DOI:** 10.2196/jmir.4943

**Published:** 2015-09-23

**Authors:** Julia M Alber, Jay M Bernhardt, Michael Stellefson, Robert M Weiler, Charkarra Anderson-Lewis, M David Miller, Jann MacInnes

**Affiliations:** ^1^ Center for Health Behavior Research Perelman School of Medicine University of Pennsylvania Philadelphia, PA United States; ^2^ Center for Health Communication Moody College of Communication University of Texas Austin, TX United States; ^3^ Center for Digital Health and Wellness Department of Health Education and Behavior University of Florida Gainesville, FL United States; ^4^ Department of Global and Community Health George Mason University Fairfax, VA United States; ^5^ University of Southern Mississippi Department of Public Health Hattiesburg, MS United States; ^6^ School of Human Development and Organizational Studies in Education University of Florida Gainesville, FL United States

**Keywords:** social media, health education, professional competence

## Abstract

**Background:**

Social media can promote healthy behaviors by facilitating engagement and collaboration among health professionals and the public. Thus, social media is quickly becoming a vital tool for health promotion. While guidelines and trainings exist for public health professionals, there are currently no standardized measures to assess individual social media competency among Certified Health Education Specialists (CHES) and Master Certified Health Education Specialists (MCHES).

**Objective:**

The aim of this study was to design, develop, and test the Social Media Competency Inventory (SMCI) for CHES and MCHES.

**Methods:**

The SMCI was designed in three sequential phases: (1) Conceptualization and Domain Specifications, (2) Item Development, and (3) Inventory Testing and Finalization. Phase 1 consisted of a literature review, concept operationalization, and expert reviews. Phase 2 involved an expert panel (n=4) review, think-aloud sessions with a small representative sample of CHES/MCHES (n=10), a pilot test (n=36), and classical test theory analyses to develop the initial version of the SMCI. Phase 3 included a field test of the SMCI with a random sample of CHES and MCHES (n=353), factor and Rasch analyses, and development of SMCI administration and interpretation guidelines.

**Results:**

Six constructs adapted from the unified theory of acceptance and use of technology and the integrated behavioral model were identified for assessing social media competency: (1) Social Media Self-Efficacy, (2) Social Media Experience, (3) Effort Expectancy, (4) Performance Expectancy, (5) Facilitating Conditions, and (6) Social Influence. The initial item pool included 148 items. After the pilot test, 16 items were removed or revised because of low item discrimination (r<.30), high interitem correlations (Ρ>.90), or based on feedback received from pilot participants. During the psychometric analysis of the field test data, 52 items were removed due to low discrimination, evidence of content redundancy, low R-squared value, or poor item infit or outfit. Psychometric analyses of the data revealed acceptable reliability evidence for the following scales: Social Media Self-Efficacy (alpha=.98, item reliability=.98, item separation=6.76), Social Media Experience (alpha=.98, item reliability=.98, item separation=6.24), Effort Expectancy(alpha =.74, item reliability=.95, item separation=4.15), Performance Expectancy (alpha =.81, item reliability=.99, item separation=10.09), Facilitating Conditions (alpha =.66, item reliability=.99, item separation=16.04), and Social Influence (alpha =.66, item reliability=.93, item separation=3.77). There was some evidence of local dependence among the scales, with several observed residual correlations above |.20|.

**Conclusions:**

Through the multistage instrument-development process, sufficient reliability and validity evidence was collected in support of the purpose and intended use of the SMCI. The SMCI can be used to assess the readiness of health education specialists to effectively use social media for health promotion research and practice. Future research should explore associations across constructs within the SMCI and evaluate the ability of SMCI scores to predict social media use and performance among CHES and MCHES.

## Introduction

### Background

Social media, or “user-generated content utilizing Internet-based publishing technologies, distinct from traditional print and broadcast media," [1] has become popular for professional, personal, and promotional use. Social media is used to connect with and communicate bidirectionally with friends, coworkers, and family [1]. Social media offers an array of tools for connecting people and sharing content, such as social networking sites (eg, Facebook and Twitter), photo-sharing sites (eg, Flickr and Instagram), and video-sharing sites (eg, YouTube and Vimeo). Compared to other types of print and broadcast media, social media is unique in that it facilitates two-way communication that allows organizations to personalize content and engage with communities and the public. Tailoring and personalizing health messages through social media can increase both the relevance of the information distributed and attention paid to the communication by the recipients [2]. Such tailoring can result in a greater impact on the intended behavior [2]. As of 2014, 74% of adult Internet users report using social media sites [3]. Thus, social media has immense potential as a medium for organizations and individuals to reach a wide range of demographic groups based on age, gender, and race/ethnicity [4].

### The Role of Social Media in Public Health Education

Social media is used to facilitate collaboration and engagement among health education professionals and the public in order to promote healthy behaviors [5-12]. Social media can engage and empower both communities and individuals to make healthier choices by helping to connect them to resources and facilitating collaboration between them to advocate for policies and programs that impact their health [13]. In a 2012 study, researchers found that approximately 60% of state health departments used at least one type of social media to meet their organizational objectives [14]. As an increasing number of health education organizations continue to take advantage of social media, use of these tools will generate numerous opportunities for influencing and changing health behavior [15-18].

Because health education specialists play a significant role in the dissemination of health information and the promotion of healthy behaviors [19], it is crucial for health education professionals to be able to capitalize upon the capabilities of different media to successfully distribute information and reach target populations [20]. The specific professional roles and duties of health education specialists are described in the document, *Seven Areas of Responsibility and Competencies for Health Education Specialists* [19]. Certified Health Education Specialists (CHES) and Master Certified Health Education Specialists (MCHES) are health education specialists who have successfully passed the CHES or MCHES examination. These examinations, administered by the National Commission for Health Education Credentialing, Inc (NCHEC), are competency-based assessments of the knowledge, application, and understanding of the *Seven Areas of Responsibility* [21]. The CHES examination reflects the entry-level sub-competencies of the *Seven Areas of Responsibility*, while the MCHES encompasses both entry- and advanced-level sub-competencies [22]. The *Seven Areas of Responsibility* provides a foundation of competencies that CHES and MCHES can use to effectively learn and apply social media technology for health education research and practice. Many of the responsibilities outlined in this document can be carried out through the use of social media. For example, *Area of Responsibility VI*, *Competency 6.1: Obtain and Disseminate Health-Related Information*, could be carried out by using Twitter or another social media platform to disseminate health information to a particular population [19]. Social media can be employed by health education specialists to not only provide access to reliable health information, but to also tailor and personalize health messages and content to individuals (Competency 7.2) [13,19]. Social media can also assist with empowering people to make healthier and safer decisions and facilitate participation (Competency 7.3) [13,19]. Social media can bring together members of communities (eg, diabetes patients), who may be dispersed across a city, a state, a nation, or the world, to provide mutual support and to work toward a common solution (Competency 2.1) [13,19]. One well-known application of social media for health promotion is the Centers for Disease Control and Prevention’s (CDC) *Tips From Former Smokers* campaign. This campaign used Web-based videos, buttons and badges, images, and podcasts to share real-life stories of individuals living with smoking-related health issues. As a result of this campaign, free smoking cessation resources were disseminated to users, an estimated 1.64 million Americans attempted to stop smoking, and 6 million nonsmokers discussed the dangers associated with smoking with friends and family [23].

While there are commonly accepted principles that guide social media use and training for public health practitioners [13,24], there are no existing standardized measures of social media competency among health education specialists. Korda and Itani [15] stressed that social media implementation “require[s] careful application and may not always achieve their desired outcomes.” Although public health research has illustrated promising applications of social media in practice [25], there are potential dangers or issues associated with using social media for health communication, such as sharing of misleading or inaccurate information or the violation of the privacy of clients or research participants [26]. Prior research has explored organizational uses of social media in health education settings [14,27,28]. Moreover, guidelines and best practices exist for planning, implementing, and evaluating social media activities in public health [5,13,24,29-31], but there is no research that has measured training or educational needs for health education professionals who are increasingly using social media to satisfy their occupational responsibilities. For this purpose, the objective of this study was to design, develop, and test a social media competency inventory for CHES and MCHES. The intended use of the inventory was for the assessment of workforce needs to inform the development of future training, educational programs, and organizational policies.

## Methods

### Overview

**Figure 1:**
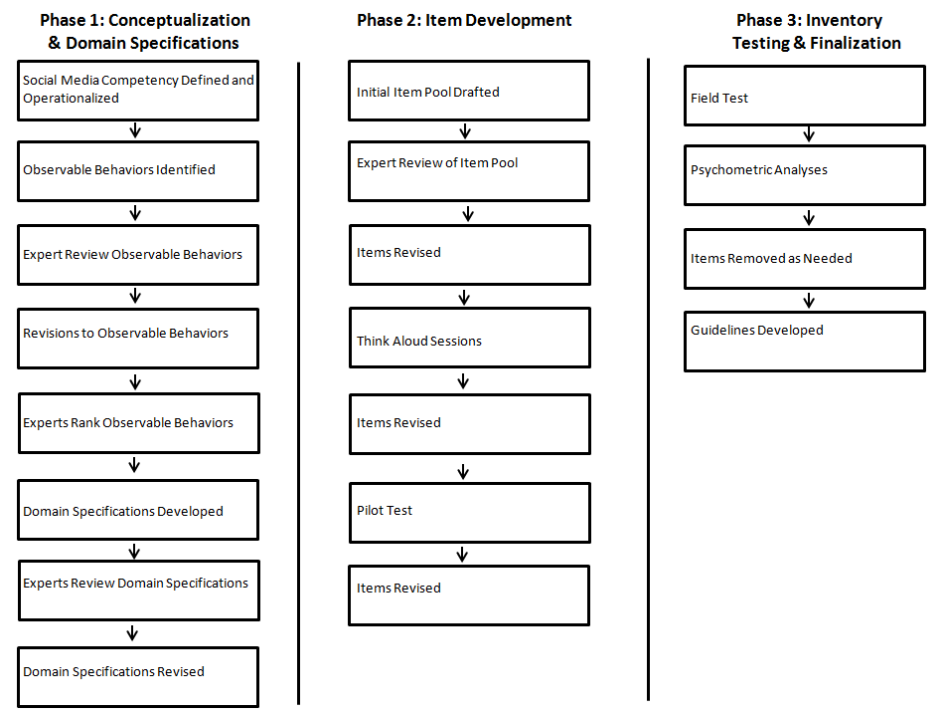
Outline of methods for designing, developing, and testing the Social Media Competency Inventory.

The design, development, and testing of the new measure for social media competency entailed three overarching phases: Phase 1, Conceptualization and Domain Specifications; Phase 2, Item Development; and Phase 3, Inventory Testing and Finalization. These phases and their corresponding steps were based on Crocker and Algina’s 10-step process of test construction [32]. Approval from the lead researcher’s (JA) university Institutional Review Board was obtained prior to beginning this study. Figure 1 depicts a sequential overview of the research activities that occurred within each of the three phases.

### Phase 1: Conceptualization and Domain Specifications

#### Defining and Operationalizing Social Media Competency

The term “social media competency” was not previously defined in the literature; therefore, a review of the literature was conducted using Google Scholar, PubMed, the Cumulative Index to Nursing and Allied Health Literature (CINAHL), and the Education Resources Information Center (ERIC) using a combination of the following keyword search terms: competency, competency model, competence, competency framework, professional ability, successful use, performance, professional readiness, employee, information technology, social media, social network, Web-based technologies, new media, digital health, technology, Web 2.0, and eHealth.

Once social media competency had been defined and operationalized, observable behaviors that characterized the specific constructs to be measured in the inventory were identified using the literature and professional guidelines. More specifically, the following terms were searched on three databases (Google Scholar, PubMed, and CINAHL): health education, health promotion, health behavior, prevention, use, guides, practice, research, competency, ability, knowledge, attitudes, readiness, effective use, information technology, social media, social network, Web-based technologies, new media, digital health, technology, Web 2.0, and eHealth. Additionally, leading health organizations’ websites were searched for guidelines and recommendations related to the responsibilities of health education specialists and evidence-based social media practices. Organizations included the American Public Health Association (APHA), Centers for Disease Control and Prevention, National Institutes of Health (NIH), National Commission for Health Education Credentialing, Inc, the Society for Public Health Education (SOPHE), and the US Department of Health & Human Services (HHS). Using these sources, a list of potential and actual social media tasks completed by health education specialists was drafted. These tasks were compared with the entry-level sub-competencies outlined in the *Seven Areas of Responsibility*. Sub-competencies that could be met using social media were revised to incorporate social media. For example, one sub-competency is related to collecting primary health data (Sub-competency 1.3.1) [19]. Using this sub-competency, the following observable behavior was created: “Collect primary data (using survey or other method to collect data directly from social media) related to health using social media.” As the inventory was intended for both CHES and MCHES, only entry-level sub-competencies were included in the development of the observable behaviors. The observable behaviors were organized according to each of the *Seven Areas of Responsibility for Health Education Specialists* [19]. This organization system was used to ensure that each of the seven areas would be adequately reflected in the final inventory, thus allowing the inventory to be directly linked to the key responsibilities of health education specialists.

#### Observable Behaviors: Expert Review and Revisions

A panel of four experts was asked to review the initial list of observable behaviors. The panel included three content experts and one measurement expert. The three content experts had worked in the field of health education for a minimum of five years, and had extensive knowledge on using social media technologies in health education research. One of the content experts is also an MCHES. The measurement expert was a research methodologist with vast experience in psychometrics and large-scale measurement and evaluation. The experts were sent a Web-based survey using Qualtrics survey software. The Web-based survey contained observable behaviors organized by the *Seven Areas of Responsibility for Health Education Specialists*. Experts were asked to indicate which behaviors should be kept or removed. Provided textboxes allowed experts to suggest additional observable behaviors for consideration.

After the initial expert review, the list of observable behaviors was revised and sent back to the experts. Experts were then asked to create a rank order of the remaining observable behaviors in each of the *Seven Areas of Responsibility for Health Education Specialists*. SPSS version 22.0 was used to calculate median ranks and interquartile ranges to determine the most important observable behaviors ranked by the experts.

#### Domain Specifications for Each Scale

Domain specifications were developed for each of the constructs identified as a function of the literature and expert review. For each construct, a table of specifications was developed to outline the content areas, the relevant learning domain levels (ie, stages of the affective domains, levels of the cognitive domain), and the representation of each of these elements across the scale. The domain specifications for each construct were again sent to the experts to review using a Web-based survey programmed into Qualtrics. Domain specifications were revised based on the experts’ comments and feedback.

### Phase 2: Item Development

#### Overview

Development of the items, item stems, and instructions were guided by recommendations from several survey methods resources [33-36]. An initial list of items was drafted using the list of observable behaviors and the representation of items outlined in the domain specifications. The number of items was based on the proportion of items in each cell of the table of specifications per construct, and by approximately doubling the number of items needed for the final scale for each construct [37].

#### Expert Review of Items and Item Revisions

Experts were asked to review items using a Web-based survey administered on Qualtrics. Within the survey, the experts were asked to evaluate the following characteristics by whether or not each item adequately reflected each characteristic (yes/no): brevity, focus, clarity, assurance, readability, and adequacy of response options [33,38]. Experts were provided with the definition of each of these characteristics. If an expert selected “no” for any item criteria, they were asked to explain why using a textbox provided below each item. Experts were also asked if any of the items could be perceived as biased or leading and were again given the opportunity to make comments in a textbox. Next, using the domain specifications for each construct as a reference, experts were asked to respond to the following question: “Overall, would you say the items in this section are representative of the universe of all possible questions related to [social media use in the health education construct]?” Finally, experts were provided the opportunity to make suggestions or propose changes related to the items and instructions included in the item pool. The items and the instructions were revised based on the feedback obtained by the experts.

#### Think-Aloud Sessions

Think-aloud sessions were conducted with CHES/MCHES using the revised item list. A think-aloud session is a type of cognitive interviewing method commonly employed for pilot-testing instruments to better understand the mental processes participants use to answer items [39]. A think-aloud session invites the participants to describe their thought processes aloud when responding to questions or reading instructions [39]. A purposive sample of CHES and MCHES (n=22) were invited to participate in the think-aloud sessions via LinkedIn [40], a professional social networking site [41-43]. To identify potential participants, the lead researcher (JA) entered “CHES" and "MCHES” into the LinkedIn search bar and invited all professionals (n=22) who appeared in the search results using either their listed email addresses (n=14) or the LinkedIn mailbox tool (n=8). Think-aloud sessions were completed over the phone (n=5) or in person (n=5). Participants were asked to open the Web-based survey containing the revised item list, read the items, and speak aloud as they responded to each item, describing how they came to the decision to answer each question. In addition, participants were asked if they had problems answering the items, if any of the questions were frustrating or confusing, or if any questions could be perceived as offensive. Each session lasted approximately 45 minutes. While the sessions were not recorded, detailed notes were taken during each session. The participants did not receive an incentive for participating in a session. A thematic analysis [44] of the qualitative data from the think-aloud sessions was used to identify the reoccurring problems or issues experienced by participants when completing the assessment. The instrument was revised based on findings from the thematic analysis.

#### Pilot Test and Revisions

A random sample of CHES and MCHES (n=400) were emailed a link to the Web-based survey. The pilot-test data were analyzed using SPSS version 22.0 with listwise deletion used for handling missing data. Cronbach alpha values were calculated for each scale of the inventory to provide a measure of internal consistency for each construct [45]. Bivariate Spearman rank correlations (ρ) were calculated to assess associations between items in each scale to identify any extremely high correlations (ρ>.90). When an extremely high correlation was found between items, items were considered to be repetitive and unnecessary and thus removed from the pool [46]. The frequency of response options was explored to determine if each response option was being used in each scale. To examine item discriminations, corrected item-total correlations (r) were computed for each item within each scale [47]. The instrument was again revised using the results from Spearman rank and item-total correlation analyses.

### Phase 3: Inventory Testing and Finalization

#### Field Test

Participants were recruited from NCHEC’s database of CHES and MCHES (N=10,073). A random sample of CHES and MCHES (n=1000) from the database were sent the link to the instrument embedded in a Web-based survey via a mailed letter and in an email. Three emails were also sent to each participant reminding them to complete the survey at their earliest convenience. Participants were given US $1.00 in the mailed letter, as this has been shown to increase response rates in Web-based survey research [34]. The first 100 participants to complete the survey also received a US $10.00 Amazon gift card. The Web-based survey included the final items on the instrument, as well as demographic and organizational items. Demographic questions on age, sex, race and ethnicity, highest degree obtained, and household income were adapted from the Behavioral Risk Factor Surveillance System (BRFSS) questionnaire [48]. Organizational questions were adapted from items used in another study of CHES assessing Internet and social media access at work and years of experience in the health education profession [27].

#### Psychometric Analyses and Item Removal

The analyses of field data were conducted using three procedures: (1) classical test theory procedures, (2) factor analyses, and (3) Rasch analyses. Each construct in the instrument was analyzed separately.

Classical test theory procedures were executed using SPSS version 22.0. For each scale, bivariate interitem correlations, corrected item-total correlations, and Cronbach alpha statistics were computed [47]. Items with corrected item-total correlations below .30 were removed [49]. For each scale, Cronbach alpha was compared to the generally acceptable standard of .70 or higher [45].

Using Mplus Editor 7, categorical confirmatory factor analyses (CCFAs) were conducted for each construct’s data to examine fit to a unidimensional model. CCFAs were conducted using weighted least-squares means and variance adjusted (WLSMV), an estimator suggested for noncontinuous data that is robust for nonnormal data [50]. Acceptable model fit is indicated when comparative fit index (CFI) values are greater than .90, root mean square error of approximation (RMSEA) values are .05 or less, chi-square test of model fit values are not statistically significant, and Tucker-Lewis index (TLI) values are less than .90 [51]. Items with low R^2^ values (variance explained) or low parameter estimates were removed from the instrument. In this study, CCFA model fit was examined by (1) checking model fit indices (eg, CFI and TLI), (2) ensuring statistically significant (*P*<.05) parameter estimates for the path of the specified model, and (3) confirming that the magnitude of the parameter estimates are consistent with the theorized model [52].

Following CCFA, the data were analyzed under a Rasch framework, specifically the rating scale model (RSM), using the computer program jMetrik [53]. The RSM tests the probability that a person with a particular ability level will select a particular category (or response option) given a specific threshold and item difficulty level [54]. The assumptions of RSM are local independence of items, unidimensionality, and monotonicity [54]. For Likert-type data, RSMs are commonly applied [53], particularly when selecting higher response options is believed to correspond to higher ability, and that the probability of moving from one option response to the next is the same relative to item difficulty across the items [55]. RSMs can reduce the number of estimated parameters compared to less constrained models (eg, partial credit model), can assist in reducing a large number of items originally developed for a scale, and requires a lower sample size than some alternative models. As the instrument developed included six different constructs, six RSMs were fitted to the data: one RSM for each of the scales.

Local independence, monotonicity, item and category infit or outfit, item difficulty, item characteristic curves (ICCs), item reliability, item separation indices, and threshold values were all examined. Local independence was investigated by examining the correlations between the item residuals [56]. Bivariate correlations between item residuals are recommended to be below |.20|, however, these correlations should be considered relative to all correlations [57]. Monotonicity was assessed by examining ICCs and determining if the threshold values increased in order (ie, higher response options had higher threshold values). Threshold values were examined to see whether or not they increased in order, and whether or not the distance between each threshold was between 1.00 and 5.00 logits [58]. For each item’s ICC, the curve that represented the lowest category was checked to ensure it was the furthest to the left, and the curve that represented the highest category was furthest to the right. Each category (or response option) was examined to ensure it had the highest probability for being observed at some point on the latent continuum. Item reliability, which provides an estimate for the quality of the item placement within an order of items along the latent trait, should have a value of .80 or greater [59]. Item separation, which provides an estimate of the quality of being able to locate items on the latent trait, should be 2.00 or greater [53,55]. Items with outfit and infit values more than 0.50 logits outside of the recommended values of 0.50 to 1.50 logits were removed from the instrument [58].

#### Guidelines Development

The reliability and validity evidence obtained during the preceding steps was used to establish general guidelines for administering, analyzing, and interpreting the final inventory. An evaluation was conducted to examine evidence of construct validity, internal structure, response process validity, external validity, and predictive validity of the inventory. Scoring and interpretation guidelines were created using recommendations from Crocker and Algina [32] and Osterlind [60].

## Results

### Phase 1: Conceptualization and Domain Specifications

#### Overview

Based on the review of literature and professional guidelines, social media competency was defined, in the context of health education, as, “the user’s potential to apply social media technologies to disseminate health information and messages, engage and empower individuals to make healthier decisions, and encourage conversation and participation related to the mission of their health organization.” Six core constructs were identified as important for assessing social media competency: (1) Social Media Self-Efficacy, (2) Social Media Experience, (3) Effort Expectancy, (4) Performance Expectancy, (5) Facilitating Conditions, and (6) Social Influence. These constructs were identified using a technology competency model framework [61] and constructs from the integrated behavioral model (IBM) [62] and the unified theory of acceptance and use of technology (UTAUT) [63]. Social Media Self-Efficacy is an individual’s confidence in their ability to use social media technologies, as a function of their employment, to meet their employer’s needs as well as to reach and engage the public. Social Media Experience includes actions or tasks completed by the individual related to social media, social media websites, and tools that exist and are utilized for professional purposes in health education. Effort Expectancy is an individual’s perceptions of the ease of using social media while at work. Performance Expectancy is one’s beliefs about the impact of social media on their job performance. Facilitating Conditions refers to an individual’s beliefs regarding the existence of technical and organizational infrastructure to support the use of social media in the workplace. Finally, Social Influence is an individual’s beliefs about how those important to them at their workplace believe they should use social media.

#### Observable Behaviors

The list of observable behaviors initially developed (n=77) were based on behaviors described in the *Seven Areas of Responsibility and Competencies for Health Education Specialists* [19], and guidelines for social media use in health promotion [13,64]. Expert panelists commented on the wording of the behaviors (eg, changing “select” to “identify”) and suggested behaviors that could be added to the list (eg, applying health literacy principles to social media campaigns). Based on their suggestions, the wording for 11 behaviors was modified and seven behaviors were added to the list.

#### Domain Specifications

Domain specifications were developed for each of the six constructs based on the literature and expert feedback. It was clear from the literature, as well as from expert feedback, that Social Media Self-Efficacy required the largest number of items (n=50) to adequately measure the content area and each level of the cognitive domain. Social Media Experience was viewed as the second-most important construct requiring the second-largest number of items (n=20). The content of both of these scales was represented in the *Seven Areas of Responsibility for Health Education Specialists*, and incorporated four levels of the cognitive domain—apply, analyze, evaluate, create—from revisions of Bloom’s Taxonomy of the Cognitive Domain [65]. The domain specifications for the four other constructs—Effort Expectancy, Performance Expectancy, Facilitating Conditions, Social Influence—were organized according to the five stages—receiving, responding, valuing, organizing, characterizing—in Krathwohl’s Affective Domain Taxonomy [66]. Scales for measuring each of these constructs were used in conjunction with expert feedback to conclude that 3 items could adequately measure each of these four constructs (ie, Effort Expectancy, Performance Expectancy, Facilitating Conditions, and Social Influence). In sum, the domain specifications across all scales represented 82 items.

### Phase 2: Item Development

#### Expert Review of Items and Item Revisions

The initial pool had a total of 148 items (Social Media Self-Efficacy = 91 items, Social Media Experience = 40 items, Effort Expectancy = 5 items, Performance Expectancy = 4 items, Facilitating Conditions = 4 items, and Social Influence = 4 items). All experts selected “yes,” indicating that all instructions and items were concise, clear, focused, and readable; had assurance; and displayed an appropriate number of response options. Experts also suggested edits for some of the items (n=74), which amounted to minor modifications in wording.

#### Think-Aloud Sessions

Think-aloud session participants (n=10) reported working in a diverse array of professional settings, including academia (5/10, 50%), nonprofits (3/10, 30%), a local health department (1/10, 10%), and a state health department (1/10, 10%). Five themes were identified from the qualitative data collected during think-aloud sessions: (1) definitions and terminology instruction, (2) item wording, (3) unintended thought process, (4) formatting and organization, and (5) suggested items. Identified themes informed revisions to items for clarification, revising instructions to be consistent across the inventory, reducing the number of items that appeared on each page of the survey, and organizing the items in the Social Media Self-Efficacy and Social Media Experience scales by the *Seven Areas of Responsibility for Health Education Specialists*. Finally, the midpoint (ie, neither confident nor unconfident) of the Social Media Self-Efficacy scale was removed because some participants selected this option only when they were unfamiliar with a word or phrase.

#### Pilot Test

A total of 36 out of 400 (9.0%) participants completed the pilot test. A total of 16 items were removed or revised based on data from the pilot test. None of the response options appeared to be severely skewed in one direction, and all response options were used by pilot survey participants. Within the Social Media Self-Efficacy scale, 9 items were highly correlated (ρ≥.90), suggesting that they measured similar concepts; therefore, these 9 items were removed. A total of 4 items were also removed from the Social Media Experience scale because of high correlations between items (ρ≥.90). A total of 1 item was removed from the Effort Expectancy scale because of a low corrected item-total correlation (*r*=.07), while 2 items from the Social Influence scale were revised based on comments from participants.

### Phase 3: Inventory Testing and Finalization

#### Field Test

A total of 353 individuals out of 1000 (35.30%) completed the Web-based survey during the field test. The demographic characteristics of field test participants can be found in Table 1. Approximately 16.1% (57/353) of field test participants did not provide demographic or organizational information on the survey. The majority of participants identified as female (263/353, 74.5%) with a mean age of 36.87 years (SD 11.58). A total of 60.9% identified as white (215/353), while 10.5% identified as black or African American (37/353) and 9.1% identified as multiple races (32/353). Over half of the participants (208/353, 58.9%) reported a household income of US $50,000 or more. Half of the participants reported having at least a master’s degree (176/353, 49.9%), 22.1% reported having at least a bachelor’s degree (78/353), and 11.9% reported earning a doctoral degree (42/353).

Organizational characteristics of field test participants can be found in Table 2. On average, participants had 10.03 years (SD 9.15) of experience in the health education field. Practice setting varied, with approximately one-quarter of participants indicating they worked in academia (88/353, 24.9%) and 15.6% reporting they worked for a nonprofit organization (55/353). Other settings included local government or health department (32/353, 9.1%), clinical (25/353, 7.1%), private or corporate (22/353, 6.2%), state government (17/353, 4.8%), federal government (21/353, 5.9%), health insurance (9/353, 2.5%), and K-12 education (3/353, 0.8%). The majority of participants (292/353, 82.7%) reported workplace access to the Internet, but less than half of participants (171/353, 48.4%) reported full access to all social media sites at their place of employment.

**Table 1:** Demographic characteristics of field test participants (n=353).

Demographics		n (%)
**Sex**		
	Male	33 (9.3)
	Female	263 (74.5)
	Missing	57 (16.1)
**Race/ethnicity**		
	White	215 (60.9)
	Black or African American	37 (10.5)
	Asian	5 (1.4)
	Pacific Islander	1 (0.3)
	American Indian or Alaska Native	2 (0.6)
	Hispanic, Latino, or Spanish	3 (0.8)
	Multiple Races/other	32 (9.1)
	Missing	58 (16.4)
**Income (US $)**		
	$24,999 or less	15 (4.2)
	$25,000 to $49,999	54 (15.3)
	$50,000 to $74,999	66 (18.7)
	$75,000 or more	142 (40.2)
	Don't know	10 (2.8)
	Missing	66 (18.7)
**Highest degree earned**		
	Bachelor	78 (22.1)
	Master	176 (49.9)
	Doctorate	42 (11.9)
	Missing	57 (16.1)

**Table 2:** Organizational information for field test participants (n=353).

Organizational information		n (%)
**Access to Internet at work**		
	Yes	292 (82.7)
	No	4 (1.1)
	Missing data	57 (16.1)
**Access to social media at work**		
	Full access	171 (48.4)
	Limited access	69 (19.5)
	No access	50 (14.2)
	Not sure	7 (2.0)
	Missing	56 (15.9)
**Employer monitors/blocks websites**		
	Yes	175 (49.6)
	No	89 (25.2)
	Don't know	32 (9.1)
	Missing	57 (16.1)
**Setting**		
	State government/health department	17 (4.8)
	Local government/health department	32 (9.1)
	Clinical	25 (7.1)
	Academia	88 (24.9)
	Nonprofit	55 (15.6)
	Private or corporate	22 (6.2)
	Federal government	21 (5.9)
	Health insurance	9 (2.5)
	K-12 education	3 (0.8)
	Other	26 (7.4)
	Missing	55 (15.6)

#### Psychometric Analyses and Item Removal

The initial classical test theory analyses revealed Cronbach alphas ranging from .64 to .99. Two alphas for data collected using the Facilitating Conditions and Social Influence scales were slightly below the .70 recommended value [45]. A total of 13 items that were highly correlated and measured similar content in two other scales were removed (Social Media Self-Efficacy = 12 items, Social Media Experience = 1 item removed). Further, 4 total items with low corrected item-total correlations (r<.30) were removed (Effort Expectancy=2 items, Facilitating Conditions =1 item, Social Influence=1 item). Analyses of the final inventory items revealed internal consistency ranging from .66 to .98, and corrected item-total correlations ranging from .41 to .86. Table 3 lists summary statistics generated from the final classical test theory procedures.

**Table 3:** Summary statistics from classical test theory procedures across final scales.

Scale	Cronbach alpha	Corrected item-total (r) range
Social Media Self-Efficacy	.98	.66-.86
Social Media Experience	.98	.75-.85
Effort Expectancy	.74	.51-.63
Performance Expectancy	.81	.60-.73
Facilitating Conditions	.66	.57-.70
Social Influence	.66	.41-.57

Initial CCFAs for the scales revealed statistically significant chi-square test of model fit indices across the scales. RMSEA values were above the recommended .05 level, aside from one scale—Social Influence—which had an RMSEA value of .04. However, many scales (n=4) were close to the cutoff value for mediocre fit (.10) [50]. With the exception of Effort Expectancy, all other TLI and CFI values were .90 or greater, indicating acceptable fit [50]. All standardized loadings were significant, ranging from .36 to .70. Only one scale (Facilitating Conditions) had standardized loadings below .50. CCFAs were conducted a second time for scales that had item(s) removed as a result of the RSM analyses. A summary of the CCFAs for each of the final scales is presented in Table 4.

**Table 4:** Summary statistics from CCFAs^a^ across final scales.

Scale	χ^2^ (df)	RMSEA^b^ value (95% CI)	TLI^c^	CFI^d^	Standardized loading range	R^2^ value range
Social Media Self-Efficacy	7376.1 (1595)^e^	.11 (.11-.11)	.91	.97	.72-.92^e^	.52-.85
Social Media Experience	1161.0 (170)^e^	.14 (.13-.15)	.96	.97	.80-.92^e^	.65-.85
Effort Expectancy	89.9 (1)^e^	.55 (.45-.65)	.63	.88	.70-.73^e^	.49-.54
Performance Expectancy	32.9 (1)^e^	.33 (.24-.43)	.94	.98	.85-.88^e^	.72-.77
Facilitating Conditions	7.1 (1)^e^	.14 (.06-.25)	.97	.99	.36-.70^e^	.13-.50
Social Influence	1.4 (1)	.04 (0-.16)	.99	.99	.52-.57^e^	.28-.32

^a^Categorical confirmatory factor analysis (CCFA).

^b^Root mean square error of approximation (RMSEA).

^c^Tucker-Lewis index (TLI).

^d^Comparative fit index (CFI).

^e^ *P*<.001.

RSM analyses were conducted initially for all scale items remaining after classical test theory procedures. Items with item infit and outfit values drastically outside of the recommended range (ie, more than 0.50 logits outside of 0.50-1.50) were removed from the Social Media Self-Efficacy scale (n=1). A review of domain specifications and item fit statistics led to the removal of items measuring similar content with worst fit statistics (Social Media Self-Efficacy=9 items, Social Media Experience=8 items, Social Media Effort Expectancy= 2 items). Initial RSM analyses showed that almost all scales possessed appropriate category fit statistics, and acceptable threshold values that increased in the appropriate order. Only one scale*,* Effort Expectancy, revealed a noteworthy issue with regard to the category thresholds. While the threshold values for the Effort Expectancy scale ranged from -2.76 to 2.33, the values did not increase in order. The original categories were 0 (Strongly Disagree), 1 (Somewhat Disagree), 2 (Neither Agree or Disagree), 3 (Somewhat Agree), and 4 (Strongly Agree). The threshold for category 2 (Neither Agree or Disagree) was larger than for category 3 (Somewhat Agree). This result presented an issue with the scale as it indicated that higher response categories do not necessarily respond to higher ability level. A follow-up RSM analysis was completed to determine if merging the neutral category (Neither Agree or Disagree) with one of the other categories would cause the thresholds to increase monotonically with either of these changes [67]. Therefore, rescoring included recoding the value of 2 to 1 (ie, scoring sequence 01123) and then recoding the value of 2 to 3 (ie, scoring sequence 01223). This allowed for the neutral category to first become collapsed with Somewhat Disagree, and then, in the second analysis, become collapsed with Somewhat Agree. Items were reverse coded before the analysis to account for the collapsed categories in each analysis. Both analyses resulted in monotonically increasing thresholds. The differences in the increased thresholds for the first change (ie, collapsing with Somewhat Disagree) were more severe than for the second change (ie, collapsing with Somewhat Agree) as evidenced by the curves in the ICCs.

Table 5 lists summary statistics for the second RSM analyses conducted for the final scale items. Item reliabilities were above the recommended value of .80 or greater [59]. Likewise, the item separation indices for each scale were above the cutoff value of 2.00 [53,59]. Almost all scales had item infit and outfit values within the recommended range of 0.50 to 1.50 logits [58], with the exception of Facilitating Conditions, which had values above 1.50 yet below 2.00 logits. Similarly, the Facilitating Conditions scale had some category infit and outfit values outside of the range of 0.50 to 1.50 logits. Some evidence of local dependence was observed across the scales with residual correlations above the recommended value of *r*=|.20|.

**Table 5:** Summary statistics from rating scale model analyses across scales of final inventory.

Scale	Item reliability	Item separation index	Item infit range	Item outfit range	Category infit range	Category outfit range
Social Media Self-Efficacy	.98	6.76	0.63-1.45	0.64-1.62	0.94-1.06	0.90-1.08
Social Media Experience	.98	6.24	0.77-1.45	0.74-1.43	0.88-1.25	0.83-1.24
Effort Expectancy	.95	4.15	0.86-1.15	0.89-1.14	0.86-1.15	0.89-1.14
Performance Expectancy	.99	10.09	0.85-1.35	0.74-1.29	0.93-1.10	0.87-1.15
Facilitating Conditions	.99	16.04	0.78-1.78	0.66-1.86	0.71-1.78	0.66-1.86
Social Influence	.93	3.77	0.88-1.16	0.86-1.10	0.83-1.11	0.78-1.08

### Final Social Media Competency Inventory

The final Social Media Competency Inventory (SMCI) can be found in Multimedia Appendix 1. The scale consists of 82 items distributed across six scales: Social Media Self-Efficacy (n=50), Social Media Experience (n=20), Effort Expectancy (n=3), Performance Expectancy (n=3), Facilitating Conditions (n=3), and Social Influence (n=3). Guidelines for the administration, scoring, and interpretation of the SMCI can be found in Multimedia Appendix 2.

## Discussion

### Principal Findings

Through a multistage instrument-development process, the SMCI was designed to measure six core constructs: (1) Social Media Self-Efficacy, (2) Social Media Experience, (3) Effort Expectancy, (4) Performance Expectancy, (5) Facilitating Conditions, and (6) Social Influence. Using a random sample of CHES/MCHES, evidence of generalizability was provided. Furthermore, including both CHES and MCHES as study participants allowed the reliability and validity evidence to be expanded to a larger population of health education specialists. The demographic and organizational data reported in the field test was comparable to recent studies including samples of CHES/MCHES [27,68].

Overall, adequate reliability and validity evidence supported the utility of the SMCI for assessing health education specialists’ use and access to social media technologies for health promotion research and practice. Furthermore, the use of think-aloud sessions during the pilot test provided response process validity evidence within the SMCI’s intended population. Information from the think-aloud sessions assisted in determining that participants were interpreting the items and response options as intended. However, because the Effort Expectancy scale experienced disorder thresholds, additional research needs to further explore the thought process of public health education specialists when interpreting and using the Likert response options for this particular scale. It is possible that the neutral option was used as “I’m not sure” or other unintended thought processes.

Results from classical test theory, confirmatory factor analysis, and Rasch RSM procedures provided evidence to support the internal structure of the scales within the SMCI. However, two scales (Facilitating Conditions and Social Influence) revealed internal consistency values below the recommended cutoff values. Nevertheless, data collected using both of these scales generated acceptable item reliability values in the RSM analyses. Based on the CCFA and bivariate residual correlation analyses, the data collected from the Facilitating Conditions and Social Influence scales should be fitted to a more multidimensional model to determine if this allowance provides a better fit for each of the scales’ data. Future research is needed to explore the external structure of the scales included in the inventory, as well as the predictive validity of the SMCI.

### Understanding the Competency and Theoretical Frameworks of the Social Media Competency Inventory

Constructs within the SMCI were selected using a competency modeling framework and a theoretical framework based on the integrated behavioral model and the unified theory of acceptance and use of technology. According to the integrated behavioral model, there are four conditions under which a behavior is most likely to occur [62]. First, a person should have strong intention to participate in the behavior as well as the knowledge and skills to perform it. Second, there should not be any substantial environmental constraints that could prevent the behavior from being performed. Third, the behavior should be important to the person. And lastly, the person should have some prior experience performing the behavior. Similar to the health behavior theories from which the IBM was established (ie, theory of planned behavior and theory of reasoned action), intention is the most important predictor of behavior. Intention to participate in a behavior offers indication of the individual’s “perceived likelihood of performing a behavior” [62]. An individual’s behavioral intention is predicted by their personal agency, self-efficacy, and perceived norms associated with the behavior. Possessing the appropriate skills and knowledge is crucial for a person to be able to successfully perform the behavior, and previous experience with the behavior can translate to the behavior becoming habitual. As with IBM, the unified theory of acceptance and use of technology also emphasizes the significance of behavioral intention, positing that behavior is predicted by behavioral intention as well as facilitating conditions [63]. Behavioral intention is the individual’s intention to use the specific technology. Facilitating conditions refer to an individual’s beliefs in the existence of technical and organizational infrastructure to support the use of the technology. Behavior intention is predicted by effort expectancy, performance expectancy, and social influence. By blending the theoretical constructs and relationships from these two frameworks, a model for assessing social media competency as well as their relationship to social media performance was created.

Marcolin et al [61] discussed different measures related to technology-competence modeling. They identified three main outcomes related to technology-related user competence: cognitive, skill-based, and affective. Cognitive outcomes refer to the individual’s knowledge about the technology and how to use the technology. Skill-based outcomes represent the transition from knowledge to automaticity, which refers to the individual’s ability to generalize his or her knowledge to new technology-related tasks. Affective outcomes refer to the motivations and attitudes of the individual as they both pertain to user competence. An instrument attempting to model competency should measure these three outcomes.

Social media competency can be explained as a person’s intention in the sense that it indicates their readiness to access and use social media as a function of their employment. This capacity is influenced by their attitudes and beliefs related to social media: to be more specific, their beliefs on how social media use impacts their ability to perform as a health education specialist, how those important to them perceive social media use, the ease of learning how to use social media for health education, and the existing technical and organizational infrastructures for using social media at their place of employment. These perceptions related to four constructs from the UTAUT: (1) effort expectancy, (2) performance expectancy, (3) social influence, and (4) facilitating conditions. These beliefs may also correspond to behavioral and normative beliefs constructs from the IBM. Furthermore, an individual’s previous experience using social media is likely to affect their capacity to use social media.

### Limitations and Opportunities for Future Research

There are several limitations that should be addressed in future research. First, the list of CHES and MCHES from which the random samples were drawn for the pilot and field tests were not inclusive of all CHES and MCHES. Only CHES and MCHES who agreed to have their contact information distributed to researchers were included on the list. However, this contact list did contain more than 75% of all CHES and MCHES. Similarly, missing data related to demographic and organizational information limits the ability to generalize the findings from the field test to all CHES and MCHES. However, it should be noted that the majority of participants (84%) did provide this information in the field test. Similar missing data related to demographics and organizational information has been observed in other studies of CHES [68]. Not all health education specialists are CHES and MCHES; therefore, future research is needed to test the reliability of SMCI data among health education specialists who are not CHES or MCHES.

Second, data collection for both the pilot and field tests were conducted through a Web-based survey with self-report data. This may have impacted the representation of CHES and MCHES. Some invited CHES and MCHES may not have wanted to participate in a Web-based survey versus a paper-and-pencil or telephone survey. However, Web-based surveys do allow for anonymous surveys, which may have decreased socially desirable responses and offered greater privacy to participants [45]. Multiple methods for data collection on each scale should be conducted in the future to generate multitrait/multimethod matrix validity evidence. Wright [69] provides several advantages of Web-based surveys for research, including reduced time and costs. Because the pilot and field tests were international in scope, it would have been far more time consuming and expensive to have participants complete the inventory in person or by postal mail. Nevertheless, the data obtained from the Web-based surveys were self-reported, and there is no guarantee that individuals provided accurate information.

Evidence of local dependency among items in the SMCI scales’ data was another limitation. Large residual correlations may suggest the possibility of multidimensionality [70]. While some research suggests that parameter estimates of item response theory (IRT) models can be somewhat robust to minor violations of unidimensionality or local dependency [71], additional research should be done to determine if multidimensionality exists for the data collected using each of the six different scales.

One last study limitation was that only some types of validity evidence were explored in this study. Types of validity evidence in need of further exploration include divergent, convergent, predictive, and multitrait/multimethod matrix. While it is important to explore convergent and divergent relationships among constructs as well as predictive validity, this was not feasible in this inceptive instrument-development study. Adding more scales to the SMCI would have made the Web-based survey even longer, and may have reduced completion rates. Nonetheless, for the purposes of interpretation, it is important to differentiate between competency and performance, and also understand the relationship between competency and performance. Future research should examine the relationship between social media competency and performance among CHES and MCHES.

### Conclusions

The growth in the popularity and functionality of social media technologies corresponds to increasing potential for engaging and reaching specific populations for health promotion activities. While health education specialists widely use social media and general guidelines for social media use in public health are available, an assessment instrument for evaluating the potential of health education specialists to effectively use social media in the workplace was previously unavailable. The SMCI, which was developed and tested in this study, provides a unique measure to assess the capacity of health education specialists to use social media technologies. The SMCI can be applied to identify gaps in confidence and experience, as well as professional development needs within health education organizations. This data can be used to inform the development of specific guidelines, training, and policies. More research is now needed to explore the dimensionality of data collected using the SMCI. Future studies should also examine the relationship among the six constructs within the SMCI, and the ability of the SMCI to predict social media use and performance among CHES and MCHES. While the results of this study do not offer absolute support for use of the SMCI in high-stake situations (eg, employment decisions), the SMCI can be used to obtain a general understanding of the readiness of health education professionals to use social media to engage populations and deliver relevant public health messages. This study provides the necessary foundation for future research that will help ensure that the health education field is sufficiently prepared to effectively use social media to promote and protect public health.

## Appendix

Multimedia Appendix 1 The Social Media Competency Inventory.

Multimedia Appendix 2 Guidelines for administration, scoring, and interpretation of the Social Media Competency Inventory.

## Abbreviations

APHA: American Public Health Association
BRFSS: Behavioral Risk Factor Surveillance System
CCFA: categorical confirmatory factor analysis
CDC: Centers for Disease Control and Prevention
CFI: comparative fit index
CHES: Certified Health Education Specialist(s)
CINAHL: Cumulative Index to Nursing and Allied Health Literature
ERIC: Education Resources Information Center
HHS: US Department of Health & Human Services
IBM: integrated behavioral model
ICC: item characteristic curve
IRT: item response theory
MCHES: Master Certified Health Education Specialist(s)
NCHEC: National Commission for Health Education Credentialing, Inc
NIH: National Institutes of Health
RMSEA: root mean square error of approximation
RSM: rating scale model
SMCI: Social Media Competency Inventory
SOPHE: Society for Public Health Education
TLI: Tucker-Lewis index
UTAUT: unified theory of acceptance and use of technology
WLSMV: weighted least-squares means and variance adjusted
