# Supplementary material for: Designing and Testing an Inventory for Measuring Social Media Competency of Certified Health Education Specialists
Source: J Med Internet Res. 2015 Sep 23;17(9):e221. doi: 10.2196/jmir.4943 (PMC4642407; doi:10.2196/jmir.4943)
Supplement: Multimedia Appendix 1 [file jmir_v17i9e221_app1.pdf]

The Social Media Competency Inventory

Introduction and Instructions

This inventory assesses the social media competency level of health education specialists. Social media are technological tools that allow users to communicate and share content. Examples of social media technologies include Twitter, YouTube, and Facebook. Social media competency can be described as an individual's capacity to use social media for health education programs and initiatives. There are six sections in this inventory:

Section A: Social Media Self-Efficacy
Section B: Social Media Experience
Section C: Effort Expectancy
Section D: Performance Expectancy
Section E: Facilitating Conditions
Section F: Social Influence

For this inventory, you will be answering questions specific to social media use in a professional setting within the field of health education. Please read the instructions for each section carefully.

Section A: Social Media Self-Efficacy

In this section, you will be asked to review different tasks that a health education specialist may complete when developing, implementing, or evaluating a social media program organized by the Seven Areas of Responsibilities for Health Education Specialists. More specifically, you will be asked to indicate how confident you would feel completing each of the tasks presented.

Area I: Assess Needs, Assets and Capacity for Health Education

For EACH task, please indicate how confident you feel TODAY in your ability to complete the task in the field of health education.

Response Options*:* (1) Extremely Unconfident; (2) Unconfident; (3) Somewhat Unconfident; (4) Somewhat Confident; (5) Confident; (6) Extremely Confident

Items*:*

- Collect primary health-related data through survey methods using social media
- Identify instruments that can be used for collecting health-related data using social media
- Analyze the capacity within your organization for developing social media program
- Use existing theories to assess social media campaigns
- Create an assessment plan for a social media campaign
- Assess the use of social media platforms for health-related purposes in populations of interest

Area II: Plan Health Education

For EACH task, please indicate how confident you feel TODAY in your ability to complete the task in the field of health education.

Response Options: (1) Extremely Unconfident; (2) Unconfident; (3) Somewhat Unconfident; (4) Somewhat Confident; (5) Confident; (6) Extremely Confident

Items:

- Identify populations of interest to reach during a social media campaign
- Apply principles of health literacy when creating social media activities
- Develop objectives for social media campaigns
- Develop social media activities and strategies that are evidence-based to meet health education objectives
- Develop social media activities and strategies that are theory-based to meet health education objectives
- Identify resources required for implementation of a social media campaign
- Identify the factors that may hinder or foster implementation of social media activities
- Apply principles of cultural competency when creating social media pages

Area III: Implement Health Education

For EACH task, please indicate how confident you feel TODAY in your ability to complete the task in the field of health education.

Response Options: (1) Extremely Unconfident; (2) Unconfident; (3) Somewhat Unconfident; (4) Somewhat Confident; (5) Confident; (6) Extremely Confident

Items:

- Monitor the progress of social media activities
- Determine the readiness of the population of interest to implement the social media program
- Apply evidence-based strategies to social media planning
- Determine the readiness of your organization to implement the social media programs
- Use appropriate social media tools to implement the campaign
- Develop a plan of action for social media programs
- Implement plan of action for social media programs

Area IV: Conduct Evaluation and Research Related to Health Education

For EACH task, please indicate how confident you feel TODAY in your ability to complete the task in the field of health education.

Response Options: (1) Extremely Unconfident; (2) Unconfident; (3) Somewhat Unconfident; (4) Somewhat Confident; (5) Confident; (6) Extremely Confident

Items:

- Assess the validity of data collected for social media research or evaluation
- Monitor data collection progress for social media activities
- Interpret findings from data collected during social media interventions
- Apply ethical standards when developing evaluation plans for social media interventions
- Identify survey instruments for data collection in social media research or evaluation
- Communicate findings from social media interventions with key stakeholders
- Apply ethical standards when conducting social media research

Area V: Administer and Manage Health Education

For EACH task, please indicate how confident you feel TODAY in your ability to complete the task in the field of health education.

Response Options: (1) Extremely Unconfident; (2) Unconfident; (3) Somewhat Unconfident; (4) Somewhat Confident; (5) Confident; (6) Extremely Confident

Items:

- Provide expert assistance for implementing social media initiatives
- Provide expert assistance for evaluating social media initiatives
- Identify potential partnership that will assist with the social media intervention
- Explain how the goals of a social media program align with the mission and goals of your organization

Area VI: Serve as a Health Education Resource Person

For EACH task, please indicate how confident you feel TODAY in your ability to complete the task in the field of health education.

Response Options: (1) Extremely Unconfident; (2) Unconfident; (3) Somewhat Unconfident; (4) Somewhat Confident; (5) Confident; (6) Extremely Confident

Items:

- Convey the advantages of using social media
- Convey the disadvantages of using social media
- Develop training programs in social media for health educators
- Develop guidelines for evaluating social media initiatives
- Evaluate the qualification of individuals who will be assisting with social media initiatives if needed
- Identify social media resources with accurate health information
- Justify the need for social media guidelines for health educators
- Identify social media resources with relevant health information
- Convey health-related information to key stakeholders using social media
- Convey health-related information to populations of interest through social media

Area VII: Communicate and Advocate for Health and Health Education

For EACH task, please indicate how confident you feel TODAY in your ability to complete the task in the field of health education.

Response Options: (1) Extremely Unconfident; (2) Unconfident; (3) Somewhat Unconfident; (4) Somewhat Confident; (5) Confident; (6) Extremely Confident

Items:

- Use social media technologies to communicate health information with populations of interest
- Lead health-related advocacy initiatives using social media
- Engage with stakeholders in health-related advocacy using social media
- Tailor health-related social media messages to individuals
- Use social media to create opportunities for professional development
- Select appropriate health-related images to be posted on social media platforms
- Evaluate the use of social media in health-related advocacy efforts
- Identify issues that may influence the use of social media for health education

Section B: Social Media Experience

In this section, you will be asked to review a set of tasks that a health education specialist may need to complete while developing, implementing, or evaluating a social media program or for advocacy or professional development purposes.

Area I: Assess Needs, Assets and Capacity for Health Education

For EACH task, you will be asked to indicate your level of previous experience completing that task within a health education setting.

Response Options: (1) None; (2) Very Limited; (3) Some Experience; (4) Quite A lot; (5) Extensive

Items:

- Collecting health data from a social media site (e.g. Twitter, Facebook)
- Determining the quality of existing social media campaigns

Area II: Plan Health Education

For EACH task, you will be asked to indicate your level of previous experience completing that task within a health education setting.

Response Options: (1) None; (2) Very Limited; (3) Some Experience; (4) Quite A lot; (5) Extensive

Items:

- Developing social media activities that are theory-based to meet objectives
- Identifying populations of interest for social media programs in health education
- Identifying key stakeholders involved with implementing a social media program

Area III: Implement Health Education

For EACH task, you will be asked to indicate your level of previous experience completing that task within a health education setting.

Response Options: (1) None; (2) Very Limited; (3) Some Experience; (4) Quite A lot; (5) Extensive

Items:

- Creating action plan for social media
- Determining the readiness of a population of interest before implementing a social media program
- Applying evidence-based strategies within a social media plan

Area IV: Conduct Evaluation and Research Related to Health Education

For EACH task, you will be asked to indicate your level of previous experience completing that task within a health education setting.

Response Options: (1) None; (2) Very Limited; (3) Some Experience; (4) Quite A lot; (5) Extensive

Items:

- Analyzing data collected from a social media intervention
- Communicating findings from social media interventions to key stakeholders
- Explaining how the use of social media aligns with your organization's mission and goals

Area V: Administer and Manage Health Education

For EACH task, you will be asked to indicate your level of previous experience completing that task within a health education setting.

Response Options: (1) None; (2) Very Limited; (3) Some Experience; (4) Quite A lot; (5) Extensive

Items:

- Recruiting individuals to help assist with the implementation of a social media intervention
- Identifying potential partnerships to help with social media interventions

Area VI: Serve as a Health Education Resource Person

For EACH task, you will be asked to indicate your level of previous experience completing that task within a health education setting.

Response Options: (1) None; (2) Very Limited; (3) Some Experience; (4) Quite A lot; (5) Extensive

Items:

- Providing expert assistance during the implementation of social media program
- Identifying social media resources that share accurate health information
- Critiquing social media campaigns for accuracy

Area VII: Communicate and Advocate for Health and Health Education

For EACH task, you will be asked to indicate your level of previous experience completing that task within a health education setting.

Response Options: (1) None; (2) Very Limited; (3) Some Experience; (4) Quite A lot; (5) Extensive

Items:

- Critiquing social media campaigns for accuracy
- Utilizing social media technologies to communicate with the public
- Using social media to empower individuals to make healthier decisions
- Posting evidence-based health messages on social media sites
- Selecting appropriate images to be posted on health-related social media platforms

Section C: Effort Expectancy

In this section, you will be asked to read statements related to social media use in health education. You will then be asked to indicate your level of agreement with each statement.

Please read EACH statement and indicate your level of agreement to EACH statement.

Response Options: (1) Strongly Disagree; (2) Somewhat Disagree; (3) Somewhat Agree; (4) Strongly Agree

Items:

- I don't like using social media in health education because it is difficult to select appropriate social media platforms
- Identifying the appropriate social media sites for my population of interest would be difficult for me
- I think it would be difficult to determine the readiness of a population of interest for a social media intervention

Section D: Performance Expectancy

In this section, you will be asked to read statements related to social media use in health education. You will then be asked to indicate your level of agreement with each statement.

Please read EACH statement and indicate your level of agreement to EACH statement.

Response Options: (1) Strongly Disagree; (2) Somewhat Disagree; (3) Neither Agree or Disagree; (4) Somewhat Agree; (5) Strongly Agree

Items:

- Social media could improve my ability to convey health information to my populations of interest

- Social media would make it easier for me to engage with my populations of interest
- Social media is a valuable tool for health education

Section E: Facilitating Conditions

In this section, you will be asked to read statements related to social media use in health education. You will then be asked to indicate your level of agreement with each statement.

Please read EACH statement and indicate your level of agreement to EACH statement.

Response Options: (1) Strongly Disagree; (2) Somewhat Disagree; (3) Neither Agree or Disagree; (4) Somewhat Agree; (5) Strongly Agree

Items:

- I think my organization would pay for me to attend a social media training if I asked
- I think my organization would provide a social media training if I requested one
- At my place of work, I think I have access to the technologies needed to use social media (e.g., computer, Internet)

Section F: Social Influence

In this section, you will be asked to read statements related to social media use in health education. You will then be asked to indicate your level of agreement with each statement.

Please read EACH statement and indicate your level of agreement to EACH statement.

Response Options: (1) Strongly Disagree; (2) Somewhat Disagree; (3) Neither Agree or Disagree; (4) Somewhat Agree; (5) Strongly Agree

Items:

- I think my organization supports the use of social media
- I think my supervisor does not support the use of social media
- My coworkers do not like to use social media for health education
