# Supplementary material for: Designing and Testing an Inventory for Measuring Social Media Competency of Certified Health Education Specialists
Source: J Med Internet Res. 2015 Sep 23;17(9):e221. doi: 10.2196/jmir.4943 (PMC4642407; doi:10.2196/jmir.4943)
Supplement: Multimedia Appendix 2 [file jmir_v17i9e221_app2.pdf]

Social Media Competency Inventory: Guidelines for Administration, Scoring, and Interpretation

Intended Use of the Social Media Competency Inventory

The Social Media Competency Inventory (SMCI) was developed in order to identify gaps and needs for social media trainings, educational programs, and guidelines for health education specialists. This inventory is intended to be used for health education specialists, and was specifically developed for the field of health education. The reliability of this instrument in other populations outside of health education has not been evaluated.

What is Social Media Competency?

Within the context of the SMCI, social media competency is defined as the user's potential to apply social media technologies to disseminate health information and messages, engage and empower individuals to make healthier decisions, and encourage conversation and participation related to the mission of their health organization. The SMCI consists of six separate scales: (1) *Social Media Self-Efficacy*, (2) *Social Media Experience*, (3) *Effort Expectancy*, (4) *Performance Expectancy*, (5) *Facilitating Conditions*, and (6) *Social Influence*.

Social Media Self-Efficacy is defined as an individual's confidence in their ability to use social media technologies as a function of their employment to meet their employer's needs as well as to reach and engage the public.

Social Media Experience is defined as an individual's completed actions or tasks related to SM, SM websites, and tools that exist and are utilized for professional purposes in health education.

Effort Expectancy is perception of the ease of using social media for health education purposes.

Performance Expectancy is the individual's beliefs about how social media will impact their ability to do their job.

Facilitating Conditions are one's beliefs in the presence of organizational and technical infrastructure to support the use of social media for health education practice and research.

Social Influence is the individual's beliefs about how those important to them think they should use social media.

Reliability and Validity Evidence for the Social Media Competency Inventory

The SMCI was developed using the follow steps: (1) development of tables of specifications; (2) content expert reviews of domain specifications and items; (3) cognitive interviews with Certified Health Education Specialists (CHES) and Master Certified Health Education Specialists (MCHES); (4) small pilot test with CHES and MCHES; and (5) larger (n=352) field test with random sample of CHES and MCHES.

The internal consistency of field test data for each scale are as followed:

Social Media Self-efficacy	(α=0.98)
Social Media Experience	(α=0.98)
Effort Expectancy	(α=0.74)
Performance Expectancy	(α=0.81)
Facilitating Conditions	(α=0.66)
Social Influence	(α=0.66)

Limitations

The internal consistency for data produced using the facilitating conditions and social influence is marginal, and the scales require additional reliability analyses. Additionally, the *Effort Expectancy* scale needs to be tested to determine the most appropriate number of response options. Future research is truly needed to understand the relationships among the constructs and how these constructs affect social media use and performance.

Scoring of the Social Media Competency Inventory

Response option scores are provided for each section of the inventory. Use these scores to calculate total scores for each individual scale: (1) *Social Media Self-efficacy*, (2) *Social Media Experience*, (3) *Effort Expectancy*, (4) *Performance Expectancy*, (5) *Facilitating Conditions*, and (6) *Social Influence*. Each scale represents a separate construct and therefore they should not be scored together.

The following item responses should be reverse coded before analysis:

Effort Expectancy scale items that require reverse coding:

- I don't like using social media in health education because it is difficult to select appropriate social media platforms
- Identifying the appropriate social media sites for my population of interest would be difficult for me
- I think it would be difficult to determine the readiness of a population of interest for a social media intervention

Social Influence scale items that require reverse coding:

- I think my supervisor does not support the use of social media
- My coworkers do not like to use social media for health education

Total Possible Scores for Each Scale

Social Media Self-Efficacy	300
Social Media Experience	100
Effort Expectancy	12
Performance Expectancy	15
Facilitating Conditions	15
Social Influence	15

Scoring by Seven Areas of Responsibilities

Both the *Social Media Self-Efficacy* and *Social Media Experience* scales are organized by the *Seven Areas of Responsibilities for Health Education Specialists*. It is recommended that you calculate the scores from each of these areas within each of these scales to determine if a deficiency lies in a specific area or in areas.

Interpretation of Social Media Competency Inventory Scores

Social Media Self-Efficacy Scale

Higher scores can be interpreted as high confidence levels, while lower scores reflect lower confidence levels.

Social Media Experience Scale

Higher scores can be interpreted as more experience using social media for health education purposes, while lower scores reflect less experience. The items do not represent all possible experiences.

Effort Expectancy Scale

Higher scores reflect a perception that social media is easy to use, while lower scores reflect perception of social media being difficult to use.

Performance Expectancy Scale

Higher scores reflect beliefs that social media will positively impact their work, while lower scores indicate beliefs that social media will negatively impact their work.

Facilitating Conditions Scale

Higher scores indicate the belief that organization and technical structure infrastructure exists in their organization to support social media use, while lower scores indicate that they do not believe the structure exists to support social media use.

Social Influence Scale

Higher scores indicate that the individual believes those around them support the use of social media, while lower scores indicate those around them do not support the use of social media.

Administration of the Social Media Competency Inventory

The SMCI has only been tested using web-based surveys. Therefore, the reliability of the data collected from the SMCI using alternative methods is unknown.

Instructions for the SMCI and for each section of the SMCI are provided in the version of the instrument. It is recommended that each section is separated when displayed to users.

The SMCI document provides instructions, items, and response options. Items are indicated with the word "item" while the response choices for their items are indicated with "response options."
